# A Deep-Learning Model With the Attention Mechanism Could Rigorously Predict Survivals in Neuroblastoma

**DOI:** 10.3389/fonc.2021.653863

**Published:** 2021-07-14

**Authors:** Chenzhao Feng, Tianyu Xiang, Zixuan Yi, Xinyao Meng, Xufeng Chu, Guiyang Huang, Xiang Zhao, Feng Chen, Bo Xiong, Jiexiong Feng

**Affiliations:** ^1^ Department of Pediatric Surgery, Tongji Hospital, Tongji Medical College, Huazhong University of Science and Technology, Wuhan, China; ^2^ Department of Control Science and Engineering, College of Electronics and Information Engineering, Tongji University, Shanghai, China; ^3^ State Key Laboratory of Management and Control for Complex Systems, Institute of Automation, Chinese Academy of Sciences, Beijing, China; ^4^ School of Mathematics and Statistics, College of Arts and Sciences, Wuhan University, Wuhan, China; ^5^ Department of Forensic Medicine, Tongji Medical College, Huazhong University of Science and Technology, Wuhan, China; ^6^ Department of Pediatric Surgery, Fujian Medical University Union Hospital, Fuzhou, China

**Keywords:** neuroblastoma, survival, deep-learning (DL), individual therapy, transcriptome

## Abstract

**Background:**

Neuroblastoma is one of the most devastating forms of childhood cancer. Despite large amounts of attempts in precise survival prediction in neuroblastoma, the prediction efficacy remains to be improved.

**Methods:**

Here, we applied a deep-learning (DL) model with the attention mechanism to predict survivals in neuroblastoma. We utilized 2 groups of features separated from 172 genes, to train 2 deep neural networks and combined them by the attention mechanism.

**Results:**

This classifier could accurately predict survivals, with areas under the curve of receiver operating characteristic (ROC) curves and time-dependent ROC reaching 0.968 and 0.974 in the training set respectively. The accuracy of the model was further confirmed in a validation cohort. Importantly, the two feature groups were mapped to two groups of patients, which were prognostic in Kaplan-Meier curves. Biological analyses showed that they exhibited diverse molecular backgrounds which could be linked to the prognosis of the patients.

**Conclusions:**

In this study, we applied artificial intelligence methods to improve the accuracy of neuroblastoma survival prediction based on gene expression and provide explanations for better understanding of the molecular mechanisms underlying neuroblastoma.

## Introduction

Neuroblastoma, arising from the developing sympathetic nervous system, is the most common form of malignancy in children (1). Although diverse treatments have been developed for different stages of neuroblastoma, survival rates only improved in low and intermediate risk patients (2, 3). Whole genome sequencing and RNA sequencing (RNA-seq) delineated the genomic and transcriptomic traits of neuroblastoma, in which *MYCN* amplification, *ALK* mutations, *PHOX2B* mutations, *TERT* rearrangements, abnormally expressed microRNAs (miRNA) such as Mir17-92a, etc., occur mostly (4–6). Utilizing these data, a number of previous studies attempted to quantitatively predict outcomes for neuroblastoma patients. For instance, chromosomal gain or loss status were used to construct a cox regression model in one attempt, whereas most studies implemented gene expression data into multivariable score models (7, 8).

In recent years, machine-learning (ML) has been widely applied in medical sciences, especially in radiography, healthcare monitoring and genomics (9–12). ML was adopted to predict the outcomes and survival time by different approaches, such as Artificial Neural Network, Supported Vector Machine, Decision Tree and so on in many types of cancer (13–15), a vast majority of which outperformed the traditional cox regression models.

Deep-learning (DL) is a subdiscipline of ML that allows computers to transform raw data through multiple levels of representations. DL-based image detection has been widely studied in the diagnosis of diabetes and cancers (16, 17). In genomics, a multilayer perceptron could predict survival in an unsupervised or supervised way and was extended in lung cancer and hepatocellular carcinoma (18–20). DL-models were also utilized to predict stages and clinical outcomes in neuroblastoma (21, 22). However, reports examining the accuracy of DL-model with survival time are lacking.

Here, we developed a DL-based model to predict outcomes using gene expression matrices. First, 172 features were selected by the chi-square test between gene expression levels and patient survival outcomes in the training cohort. K-means clustering method was used to divide these gene features into two groups. A two-layer neural network decoder was then used to predict survival probabilities and status. F-score, accuracy, sensitivity and specificity were calculated to demonstrate that our model could precisely classify patients. To examine the robustness of our approach, we applied the same procedure in the validation cohort. Indeed, the area under the curve (AUC) of our model was 0.974 in the 5-year-survival receiver operating characteristic (ROC) curve, outperforming existing prognostic models. Furthermore, we partitioned the patients into two subgroups according to their feature expression levels. These two subgroups diverged in survival by log-rank test in Kaplan-Meier (KM) curve with *p* < 0.001. Gene Ontology (GO) enrichment analysis showed that the gene feature group 1 was enriched in the JAK-STAT pathway, while genes involved in bone morphogenesis were enriched in group 2. Therefore, this DL-based approach could rigorously predict neuroblastoma survivals and shine lights over the molecular mechanisms underlying neuroblastoma.

## Materials and Methods

### Data Acquisition

A total of 721 microarray samples, including two datasets named GSE49710 and E-MTAB-8248 (for short, GSE49710 and EMTAB), both detected on Agilent-020382 Human Custom Microarray 44k, were retrieved from NCBI Gene Expression Omnibus and ArrayExpress. The GSE49710 cohort, part of the SEQC project, portrayed Chinese neuroblastoma atlas, while EMTAB displayed German characteristics. Gene expression matrices accompanying clinical information were downloaded directly for the following analyses. F-score, accuracy, sensitivity and specificity were calculated on the whole GSE49710 cohort. Besides, GSE49711, the RNA-seq result of the same samples from GSE49710, was also fetched for lncRNA-related analysis.

### Data Preprocessing

To reduce the biases between the two datasets, we normalized the expression levels by equation (1) since the data should be better limited in 0 to 1 in the neural network.

(1) $$f_{i}\prime =\frac{f_{i}-\min(f_{i})}{\max(f_{i})-\min(f_{i})}$$

*f_i_* here indicates the expression of each RNA. $f_{i}\prime$is designated for the transformed level.

### Feature Selection

After data normalizing, significant gene expression features were selected by chi-square test which is implemented by ‘chi2’ function in the python package *sklearn *(https://scikit-learn.org/). Genes whose FDR in the chi-square test was less than 0.05 were filtered. Following this principle, only 172 differentially expressed genes were chosen.

Another common feature selection approach, the Principal Component Analysis (PCA), was used to transfer gene expression matrix into principal components. The cox proportional regression was used to filter the components. We then compared the results of the PCA method with the chi-square method.

### Feature Classification

After feature selection, a classifier was built to classify genes into different subgroups. Genes with similar biological functions were clustered into the same group. The K-means model in *sklearn* divided the selected genes into two clusters.

### Model Construction

Then a supervised classification model based on deep neural networks was built. Both of two feature groups were used as inputs of our classification. The output of this neural network was a patient’s probability which ranged between 0 and 1. 0 would indicate that the patient is likely to be alive and 1 would indicate that the patient would probably be dead.

**Figure 1:**
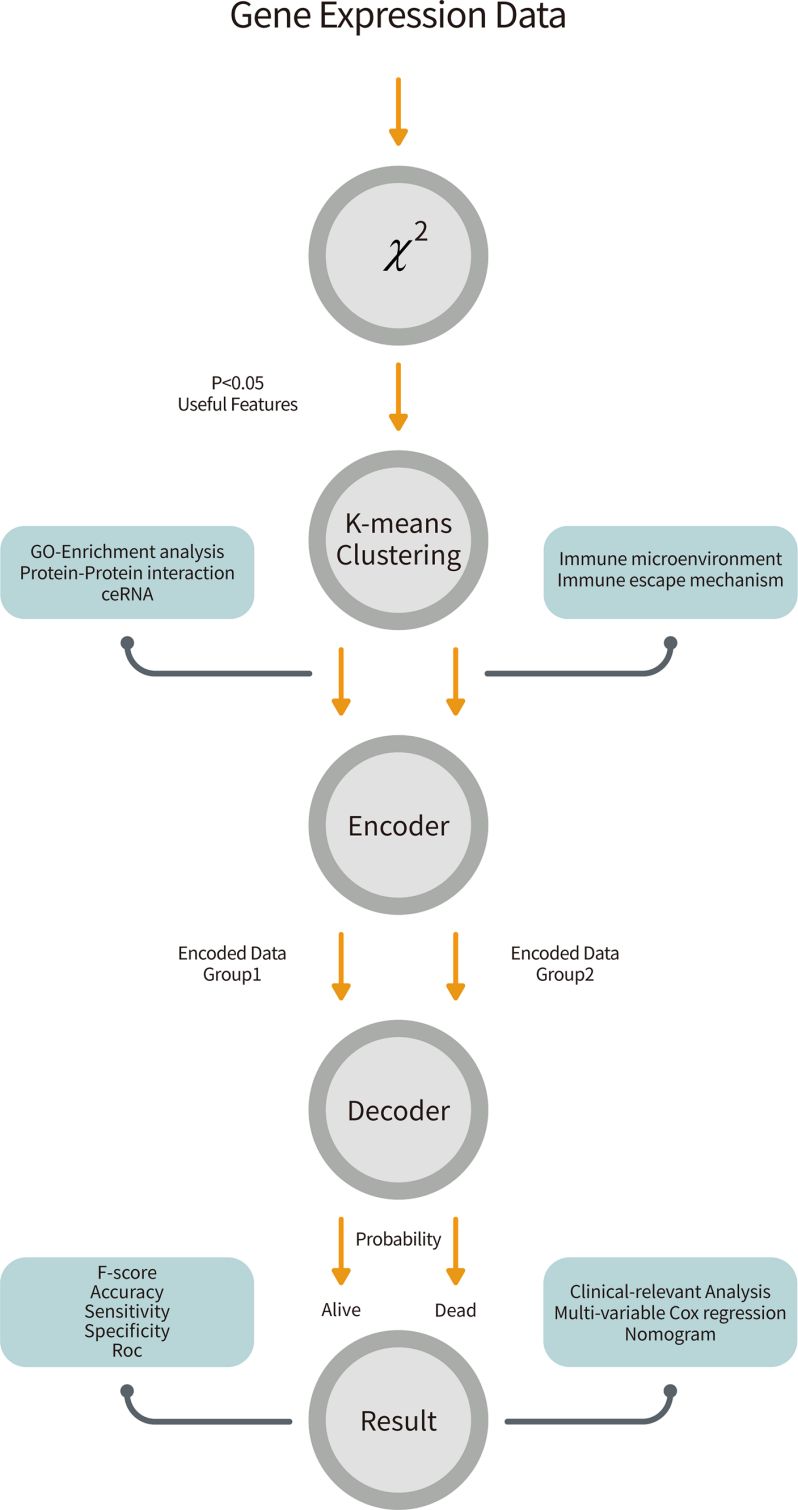
The overall workflow of our pipeline. Gene expression data from GSE49710 was retrieved and performed a chi-square test to filter 172 features. The K-means clustering method partitioned patients and genes into two groups. We trained two neural networks for two groups of features and combined them by the attention mechanism to predict survivals. Further, we analyzed biological effects between two groups and did clinical-relevant analysis.

The structure of this classifier can be seen in **Figure 1**. It consisted of two parts, the encoder and the decoder. For the encoder part, we encoded two different groups of features into two 10 dimensional features by two different two-layer networks.

(2) $$g_{1}^{\prime }=f(w_{12}f(w_{11}f_{g1}+b_{11})+b_{12})$$

(3) $$g_{2}^{\prime }=f(w_{22}f(w_{21}f_{g2}+b_{21})+b_{22})$$

$g_{1}^{\prime }g_{2}^{\prime }$ are encoded features. *w_ij_* are weights of the networks. *f_g_*_1_ and *f_g_*_2_ are transformed expressions using formula (1) and *b_ij_* are biases of the networks. The function f indicates the activation function which is a nonlinear part of the encoder. Here, we used the ReLU as this nonlinear function:

(4) f(x)={x x>00 x≤0

To combine these two different encoded features together, we applied the attention mechanism to this model (23). With the difference of simply concatenating different features, this attention mechanism can learn the relationship between them.

(5) $$G=\left[sig\left(g_{2}^{\prime }\right)*g_{1}^{\prime },sig\left(g_{1}^{\prime }\right)*g_{2}^{\prime }\right]$$

This step is illustrated in equation 5. G refers to combined features. sig is a nonlinear function.

(6) $$sig(x)=\frac{e^{-x}}{1+e^{-x}}$$

After seizing combined features, we input them into the decoder part. The decoder part is also a two-layer neural network.

(7) $$y\prime =sig(w_{32}f(w_{31}G+b_{31})+b_{32})$$

y' is the output of this classifier.

To train this network, we defined the loss function as equation 8.

(8) $$L=\{-\alpha {\left(1-y\prime \right)}^{\gamma }logy\prime y=1-(1-\alpha )y\prime {\;}^{\gamma }\log(1-y\prime )y=0$$

α and γ are parameters. In this system we set α to 0.2 and γ to 2. *y* is the true label of each patient. The patients of each group were uneven, so we used Equation 8, which is called the focal loss and was designed to solve this problem instead of cross entropy loss function (24). To avoid over-fitting, we drop 20% neurons in each layer by the dropout method. GSE49710 was chosen to train the network and EMTAB was to validate our model. For GSE49710, 70% of samples were used to train and the rest were to test.

All of the algorithms mentioned in this subsection were realized by *tensorflow* 2.2 (https://tensorflow.google.cn/). To optimize this neural network, we applied Adam optimizer and set the learning rate to 0.01 (25). The weights of networks were initialized by *glorot* uniform distribution (26).

### Model Appraisal

To further evaluate our model, we calculated the accuracy, sensitivity, specificity as well as F-score of our model in two cohorts (27).

$$Accuracy=\frac{TP+TN}{Total}$$

$$Sensitivity=\frac{TP}{TP+FN}$$

$$Specificity=\frac{TN}{TN+FP}$$

$$F1=2*\frac{Precision*Recall}{Precision+Recall}$$

ROC curves and AUCs were estimated by *pROC* package in R to assess the performance of the classifier (28). The time-dependent ROC (tROC) curve and its AUC were estimated by *survivalROC* R package to introduce survival time into our classifier (29). The curves were plotted by *ggplot2* R package (30). Besides, traditional cox regression models, devised by Zhong et al. (31) and De Preter et al. (32), were compared with ours.

### Patients Clustering

In order to correspond patients to those two groups of gene features, we performed K-means clustering on patients. *ConsensusClusterPlus* was used to determine the best k with parameters (cluster algorithmml: km, distance: Euclidean, replicate time: 1000) (33). The CDF plot and the consensus matrix instructed us to cluster patients into 2 groups. A heatmap showing expression levels of features across samples were made by *complexHeatmap* R package (34). Survival rates between these two subgroups were measured by the log-rank test in KM curves. R packages *survival* and *survminer* were used to fit KM equation and plot the curves (35, 36).

### Clinical-Relevance Analyses

In order to test whether our model was independent of other clinical factors and beneficial for clinicians to identify patients’ conditions, we first applied univariable cox regression on the age, *MYCN* status, gender, tumor stage, INSS-Risk and our probability score. A multivariable cox regression determined whether a covariate involved was decisive. Forest plots were plotted by *forestplot* package (https://CRAN.R-project.org/package=forestplot). Decision curve analysis was done by ggDCA (https://cran.r-project.org/web/packages/ggDCA/index.html). The construction and plot of the nomogram which can help clinicians to predict survival were done by *rms* (https://CRAN.R-project.org/package=rms) and *regplot* (https://CRAN.R-project.org/package=regplot) R package. Finally, an alluvial diagram was used to visualize the characteristics and disease progressions of each patient. This was achieved by the *ggalluvial* R package (37).

### Biological Function Prediction

All biological analyses were done on GSE49710. We executed GO enrichment analysis on the two groups of features by *clusterprofiler* R package respectively (38). Gene Set Enrichment Analysis (GSEA) was done by *GSEA* software (Broad Institute, Inc., version 4.0.3) with gene set ‘c5.all.v7.1.symbols.gmt’ and default parameters. *String* (https://string-db.org/) was used to identify protein-protein interactions (PPI) between the 172 features and *Cytoscape* software was used to visualize the interaction networks. *CytoHubba*, a module inside *Cytoscape*, was carried out to identify the hub genes with 12 algorithms (39, 40). For lncRNAs searching, we extracted 250 lncRNA expressions by sorting lncRNA names in *gencode.v34.long_noncoding_RNAs.gtf*, a collection of known lncRNAs downloaded from GENCODE (https://www.gencodegenes.org/). Only lncRNAs that owned a standard error > 0.2 could be enrolled in the correlation test between the 18 mRNAs. The cut-off values were: *p* < 0.05 and |coefficient|>0.5. *TarBase v.8* and *LncBase Predicted v.2* were used to query for mRNA-miRNA and lncRNA-miRNA pairs respectively (41, 42). TarBase v.8 gave 78 *CCNB1*-binding miRNAs with filters (Species: Homo Sapiens, Method Type: High-throughput, Regulation Type: DOWN, Validation Type: Direct). LncBase Predicted v.2 provided predicted lncRNA-miRNA pairs with cut-off 0.7. Finally, a competing endogenous RNA (ceRNA) network was constructed using *Cytoscape*.

### Immune Microenvironment Estimation

Inferred abundances of immune cells and normal tissue cells were calculated by single-sample gene set enrichment analysis (ssGSEA) using *GSVA* R package (43). Gene sets, also known as the markers of each cell, were collected by Charoentong et al. (44). Univariable cox proportional regression tests were exerted on all cells to reveal prognostic immune cells.

### Statistical Analysis

For categorical and continuous data with normal distribution, we applied chi-square tests and student t tests to distinguish the differences between groups. When continuous data was not normal distributed, Wilcoxon sum rank tests and ANOVA were utilized. The Pearson correlation test was used to find linear connections between two groups of observations. A *p*-value<0.05 was considered statistically significant except for emphasis. To account for multiple-testing, the *p*-values were adjusted using the Benjamini-Hochberg FDR correction. All statistical analyses were two-tailed and done by Python (Python Software Foundation, version 3.8.2) and R (R Foundation, version 3.7.0).

## Results

### Neuroblastoma Genomic Atlas Was Depicted by 172 Features

**Figure 2:**
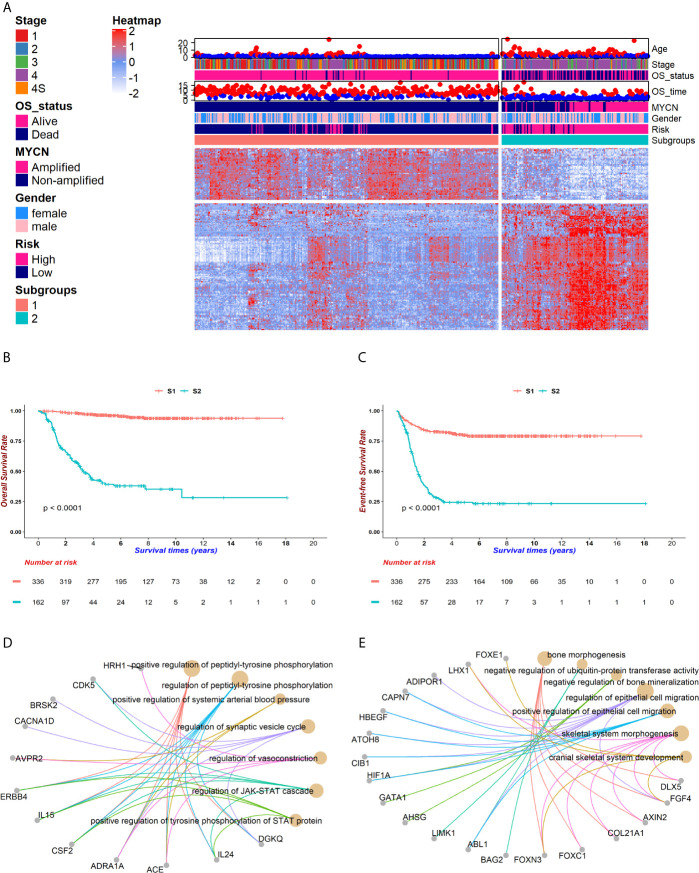
The genomic atlas of neuroblastoma was characterized by 172 genes. After implementing the chi-square test between gene expressions and survival status, a total of 172 genes were selected for following investigations. Patients were clustered into two groups, named subgroup 1 (S1) and subgroup 2 (S2), which owned 50 and 122 markers respectively. **(A)** The heatmap portraited the neuroblastoma genomic landscape in GSE49710. Gene expressions were normalized among samples. The higher expressions reached red while lower reached white. Corresponding clinical information, including age, stage, vital status, survival time, *MYCN* amplification, gender, INSS risk and subgroups, was attached on the top of the heatmap. **(B, C)** Kaplan-Meier (KM) curves showed distinct survivals between S1 (coralline line) and S2 (atroceruleous line) in overall survival (OS) **(B)** and event-free survival (EFS) **(C)** (log rank test *p* < 0.001 for each). **(D, E)** GO-enrichment analysis for feature 1 (F1) and feature 2 (F2) showed that S1 was up-regulated in the JAK-STAT pathway **(D)** and S2 was up-regulated in bone morphogenesis **(E)**.

The overall workflow is shown in **Figure 1**. After implementing the chi-square test into each feature and the survival in GSE49710, 172 features were selected (**Figure 2A**). The consensus clustering method determined the best k value as 2 to partition the features based on the expression matrix (**Supplementary Figure 1**). After that, K-means method was used to cluster the features and patients into 2 groups. Fifty genes were the markers of subgroup 1 of 336 patients (for short, S1), whereas the other 122 genes were the markers of subgroup 2 of 162 patients(S2). Basic characteristics were distributed diversely between the two subgroups except for gender (**Supplementary Table 1**). It is noteworthy that no *MYCN* amplification was detected in S1 and 92 were detected in S2 in the GSE49710 cohort while only 1 such case was detected in S1 and 45 in S2 in the EMTAB cohort, suggesting that these 172 features and the corresponding subgroups were *MYCN*-relevant (chi-square test *p* < 0.001, **Supplementary Table 1**). Also, these subgroups exhibited significant differences in overall survival and event-free survival (both log-rank test *p* < 0.001, **Figures 2B, C** and **Supplementary Figure 2**).

To understand the potential biological functions of the genes in each group, we performed GO-enrichment analyses. Notably, many features in group 1 (F1) are related to the JAK-STAT signaling while features in group 2 (F2) aggregated in the cell migration, bone morphogenesis and ubiquitin-protein transferase activities (**Figures 2D, E**). The JAK-STAT pathway promotes tumor cell proliferation, invasion and immunosuppression through a membrane-nucleus cascade (45). A Previous study has shown that the JAK1/2 inhibitor, AZD1480, could abate neuroblastoma tumor cells growth and extend survivals, suggesting that S1 patients not only maintained better survivals with neuroblastoma, but also might potentially respond to drugs such as AZD1480 to recover (46). Next, the GSEA analysis revealed that S1 showed a higher level of metabolism compared to S2 and S2 developed an intensive immune response (**Supplementary Figure 3**). This might be attributed to the mild symptoms in S1 where patients kept a normal or slightly elevated metabolism. However, accompanying the progression of tumors in S2, the patients started a fierce immune reaction and finally exhausted. These findings suggest that the subgrouping method could help to understand the molecular pathology underlying the differences in prognoses of neuroblastoma patients.

### The Neural Network Model Manifested Great Performance in Classifying Neuroblastoma

An encoder-decoder model was then trained on the GSE49710 dataset to predict survivals (**Figure 1**). Since F1 and F2 contributed unequally to the body responses and outcomes, two neural networks were created for them separately in the encoder. In this encoder, a widely used activation function, the ReLU function in the hidden layer; and a binary classification function, the sigmoid function (or say logistic function), in the output layer were employed. The attention mechanism, inspired by human physiology that people would only concentrate on tasks at hand to improve the efficacy of the encoder-decoder framework with rich information, was used to combine the two encoder parts into the decoder (47). The sigmoid function was also used in the final layer, which outputted survival probabilities. If the probability is less than 0.5, we predicted this patient as alive and vice versa (**Supplementary Table 2**).

**Figure 3:**
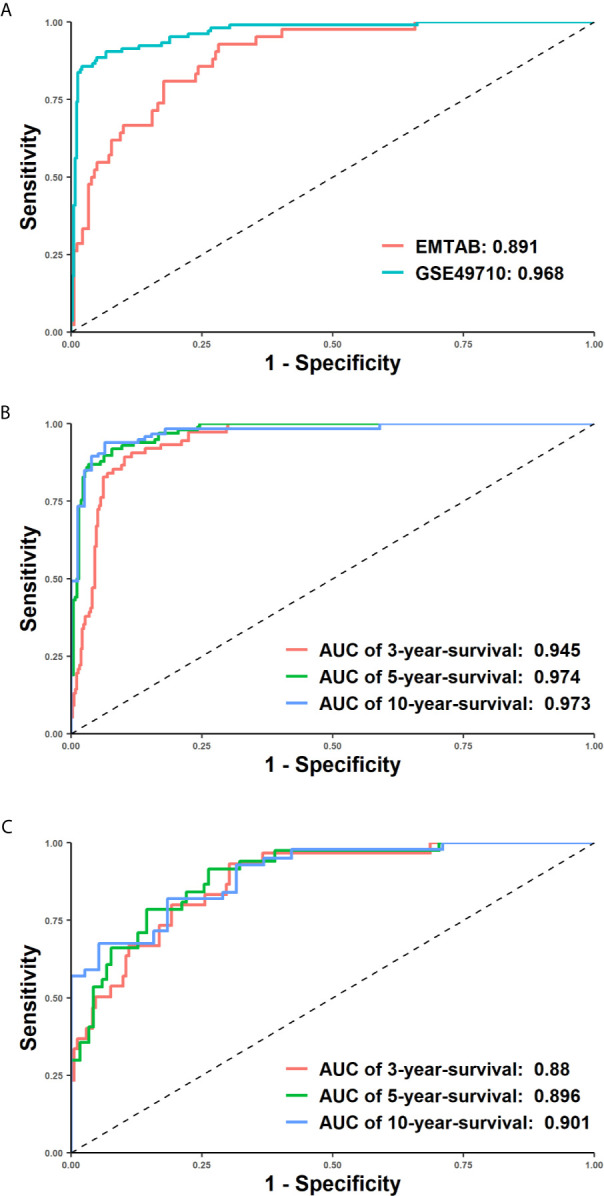
Receiver operating characteristic (ROC) curves demonstrated the superiority of our model. X-axis represented false positive rate (1-specificity) and y axis represented true positive rate. **(A)** The ROC curves of the EMTAB (coralline line) and GSE46960 (atroceruleous line) cohort. **(B, C)** 3-year, 5-year and 10-year time-dependent ROC curves of the GSE46960 **(B)** and EMTAB **(C)** cohort.

To assess the prediction quality of the overall survival status, we calculated the accuracy, sensitivity, specificity and F-score of our model, which reflected the proportion of correct predictions in all samples, true positives in all positives, true negatives in all negatives and the harmonic mean of precision and recall. In the training set, the accuracy (0.918), sensitivity (0.913) and specificity (0.944) were all greater than 0.9, suggesting that it could efficiently forecast whether a patient would be alive or dead using 172 features. Moreover, a descent efficacy was achieved in the test set (accuracy: 0.852, sensitivity: 0.911, specificity: 0.605), however, F1 score was slightly higher (GSE49710: 0.881, EMTAB: 0.886), indicating that the model was suitable for cohorts of various genetic background. The ROC and tROC curves were then generated which further demonstrate the eminence of the neural network (**Figures 3A–C** and **Table 1**). The AUCs of the training set and the test set achieved 0.968 and 0.891 respectively (**Figure 3A**), alluding the robustness of our neural network prediction model. Adding survival time into ROC curves, we found that the 5-year-survival AUCs could be boosted to 0.974 in GSE49710 (**Figure 3B**) and 0.896 in EMTAB (**Figure 3C**), which validated that our neural network could classify patients with high precision. Twenty times of 10-fold cross validation showed the stability and robustness of our neural network architecture, suggesting that our attempts of the attention mechanism would be extended into more datasets.

**Table 1:** AUCs of ROCs, 3-year-ROC, 5-year-ROC and 10-year-ROC with 20 times 10-fold cross validation.

Dataset	AUC	AUC of 3-year-ROC	AUC of 5-year-ROC	AUC of 10-year-ROC
Train	0.996 ± 0.009	0.963 ± 0.009	0.991 ± 0.006	0.993 ± 0.007
Test	0.878 ± 0.024	0.879 ± 0.028	0.907 ± 0.021	0.904 ± 0.030
Validation	0.862 ± 0.088	0.867 ± 0.076	0.895 ± 0.066	0.910 ± 0.095
EMTAB	0.865 ± 0.017	0.838 ± 0.022	0.863 ± 0.021	0.853 ± 0.032

The GSE49710 cohort was split at 7:3 into train and test cohort to perform 20 times 10-fold cross validations. In each calculation, 10% of the train cohort was randomly chosen into the validation group. The best model with the most AUC was further validated in EMTAB cohort. The probabilities of patients from the output layer were used in time-dependent ROC analyses. If the probabilities were less than 0.5, we predicted corresponding patients would be alive and if were greater than 0.5, they would be dead. These binary predictions would be compared with true labels in ROC analyses. All AUCs were expressed as mean ± standard deviation.

### Performance Comparison With Alternative Methods

**Figure 4:**
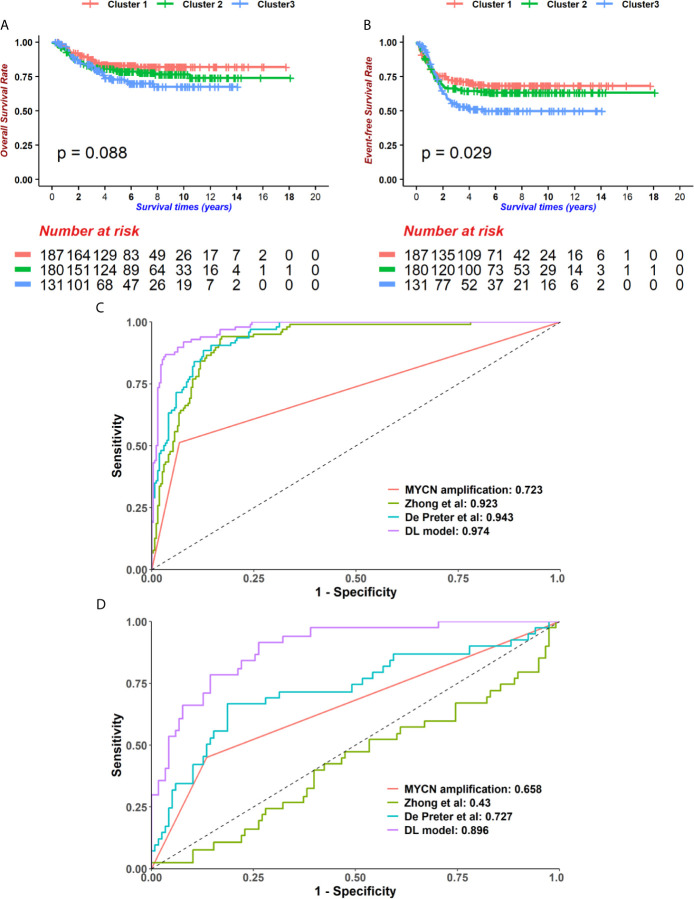
The Deep-learning-model (DL-model) outperformed alternatives in two aspects. **(A, B)** We employed the Principal component analysis (PCA) method to cut features down to 200. Using 200 PCA dimensions, we distributed patients into 3 groups, which was determined by the consensus clustering method. These groups did not show prognostic value in overall survival (OS) (**A**, log-rank test *p* = 0.088, n = 498) but event-free survival (EFS) (**B**, log-rank test *p* = 0.029, n = 498). **(C, D)** Compare with other models (*MYCN* status, 4-gene-signature by Zhong et al. and 42-gene-signature by De Preter et al.), our DL-model received the highest AUCs of 5-year-survival ROC curves in GSE49710 **(C)** and EMTAB **(D)** cohorts.

Next, we compared the performance of alternative methods which varied in either feature selection or model construction with our model. To demonstrate that our feature selection was more closely related to prognoses, a broadly used dimension reduction and feature selection method, PCA, was utilized to select features on GSE49710. Kaiser-Harris Criterion suggests that those principal components whose eigenvalue were more than 1 would be retained. In our study, all components had an eigenvalue greater than 1. Variances explained in each component were similar (**Supplementary Figure 4**). The top 200 principal components were chosen with cumulative variance percent at 89.532% for further analyses. Since the survival data has not been utilized, a univariable cox regression model was implemented to principal components, resulting in 17 components being selected with *p* < 0.05. The Consensus cluster determined the best k=value as 3 using K-means clustering (**Supplementary Figure 5**). However, the 3 subgroups were not significantly different in OS (**Figure 4A**, log-rank test *p* = 0.088) but in EFS (**Figure 4B**, log-rank test *p* = 0.029), which indicated that our chi-square-based feature selection could highlight the hub genes in neuroblastoma and partitioned patients into high and low risk groups.

We then compared the performance of our model with several existing models. In order to reduce the biases between the cohorts used in this study and in the literatures, the expression data was normalized before calculating risk scores. We selected two previously published models as well as the ‘gold standard’, *MYCN* status, and then performed survival prediction in the GSE49710 and EMTAB cohorts (**Supplementary Table 2**) (31, 32). Our DL-model generated the highest AUC in 5-year tROC curves in both GSE49710 and EMTAB cohorts (**Figures 4C, D**), indicating that our model outperformed the existing model in survival prediction.

### DL-Model Probabilities Were of Clinical Significance

**Figure 5:**
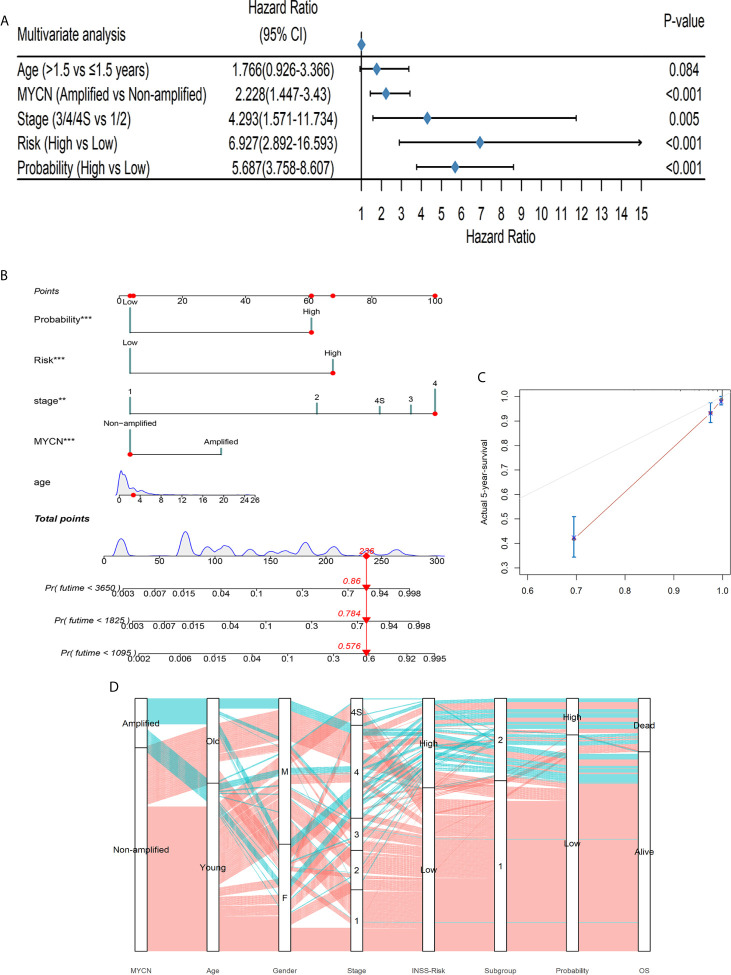
The DL-model was independent of clinical covariates and could aid to diagnose. **(A)** The DL output probability was significant in the multivariable cox regression with clinical covariates (*p* < 0.001). **(B)** A nomogram could be beneficial for survival time prediction. **(C)** The calibration curve of the nomogram with a *C*-index: 0.889. **(D)** The alluvial diagram visualized the general conditions of patients. Coralline lines represented patients without *MYCN* amplified and atroceruleous lines represented those with *MYCN* amplified.

To test whether the DL-based prediction model is widely useful for patients of various background conditions, we implemented the univariable cox regressions among the age, *MYCN* status, gender, diagnostic stage, risk and our output probability. All variables were converted to binaries in this test (**Supplementary Figure 6**). Only gender failed in this test as *p* > 0.05, which was discarded in the multivariable regression. In the multivariable cox model, the probability risk was still significant (**Figure 5A**), indicating that our DL-based model had a broad prognostic ability regardless of clinical covariates.

We further used the decision curve analysis (DCA) to evaluate the net benefit of different models (48). We constructed 3 models: only DL-probabilities, only clinical covariates in multivariable cox analysis as well as a combined model. The combined model achieved the highest net benefit no matter how risk threshold was set (**Supplementary Figure 7**). The data implied that combining DL-model and clinical information could be profitable for clinicians to diagnose and to predict survivals. Therefore, we build up a nomogram which could help clinicians to predict the potential outcomes of the patients beforehand the medical treatments (**Figure 5B** and **Supplementary Table 3**). A *C*-index 0.889 of the nomogram along with the calibration curve predicted 5-year-survival, demonstrated that this scoring system would be handy and practical in the first-line diagnosis (**Figure 5C**).

The alluvial diagram summarized the samples in our study (**Figure 5D**). 69.07% (67/97) of *MYCN*-amplified patients would be at stage 4 and this tendency was notable (chi-square test: *p* < 0.001). Only 21 patients diagnosed INSS low risk were classified into S2 and 35 with high risk into S1, showing that our prognostic subgroups were highly clinical-relevant (chi-square test *p* < 0.001). Subgroups and probabilities were also correlated (chi-square test *p* < 0.001). In the alluvial diagram, we observed that if patients had *MYCN* amplified, whether they were old or young, male or female, most of them would be at stage 4, INSS high risk, Subgroup 2. Whereas *MYCN* was not amplified, an antithetical conclusion would be drawn.

### A *CCNB1*-Associated ceRNA Network Is Related to the Survivals of Neuroblastoma Patients

**Figure 6:**
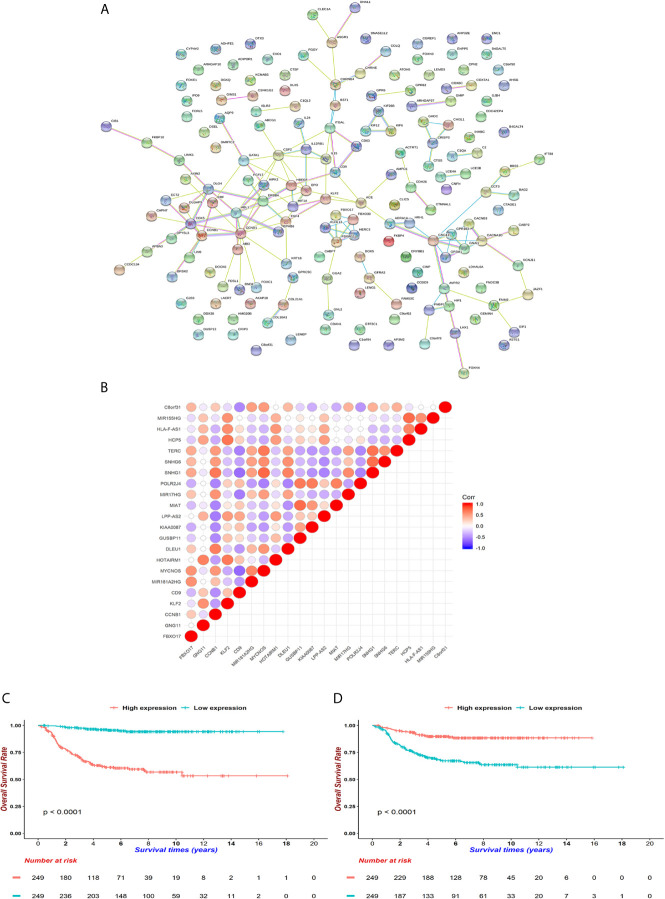
Underlying hub genes and associated interactions in neuroblastoma. **(A)** The protein-protein-interaction (PPI) network was constructed by *String* website. **(B)** Mutual correlations among 18 hub genes filtered by *cytoHubba* module in *Cytoscape* software. The dots are colored red when Pearson correlation coefficients approach 1 and dots are colored blue when coefficients reach -1. **(C, D)** KM curves for CCNB1 and CD9 **(D)**. Expressions were cut by median levels. (both log-rank test *p* < 0.0001, n = 498).

In order to isolate the hub genes of these 172 features, we first retrieved their PPI on the *String* website (**Figure 6A**). We then input them into *Cytoscape* software, and used the *cytoHubba* module to uncover hub genes by using 12 different algorithms. We summed up the top 5 genes in each algorithm and finally selected 18 genes as pivotal molecules in the network.

Next, we aimed to identify lncRNAs that could participate in the regulation of hub genes. GSE49711 is the RNA-seq of the same sample with GSE49710 and was used to uncover potential lncRNA-mRNA pairs. Subsequently, 5 mRNAs (*FBXO17*, *GNG11*, *CCNB1*, *KLF2* and *CD9*) were highly connected with 17 lncRNAs (**Figure 6B** and **Supplementary Table 4**). We noticed that both *CCNB1* and *CD9* could interact with 4 lncRNAs (*MYCNOS*, *TERC*, *SNHG1*, *MIR17HG*), along with positive coefficients between *CCNB1* and lncRNAs and opposite trends on *CD9*. *CCNB1*, an oncogene that controls the cell cycle at G2/M, had been found to be overexpressed in hepatocellular carcinoma and pancreatic cancer (49, 50). *CD9* is a tetraspanin involved in cell adhesion, metastasis and inflammation in cancer (51, 52). *CCNB1* curtailed and *CD9* increased survivals in GSE49710 and *R2* (https://hgserver1.amc.nl/cgi-bin/r2/main.cgi), which were consistent with previous reports (both *p* < 0.001, **Figures 6C, D**) (49–52).

The ceRNA theory proposed that lncRNAs and mRNAs competed to interact with shared miRNAs, up-regulating downstream RNAs by impairing miRNA activities (53). We created a *CCNB1*-associated ceRNA network as described in Methods. (**Supplementary Figure 8**, **Supplementary Table 5**). Of note, the mir-302 family (hsa-mir-302-a, -b, -c and -d) which was highly expressed in embryonic stem cells, was associated with *CCNB1* and *MIR17HG*. This indicated that mir-302 might reduce the proliferation of neuroblastoma as it did in other cancers (54, 55).

### Inhibitory Cells and Cytokines Increased in S2

Since distinct immune response patterns were observed between the two subgroups in GO-enrichment analysis and GSEA (**Supplementary Figure 3**), we further analyzed the immune microenvironment in neuroblastoma. The ESTIMATE algorithm was used to infer the purity of the microenvironment by scoring immune and stromal cells (56). Two groups did differ in stromal scores but not in immune scores, suggesting that S1 might preserve more normal stromal cells (**Supplementary Figure 9**). We used the ssGSEA algorithm to convert gene expression data into relative cell proportions (**Supplementary Figure 10A**). The numbers of the T regulatory cells (Tregs), Natural Killer (NK) cells, Monocytes, MDSCs, Eosinophils and central memory CD4 T cells were up-regulated in S2 compared to S1, while the Memory B cells, Macrophages, Gamma Delta T cells and central memory CD8 T cells exhibited opposite trends. Despite a rich amount of innate cytotoxic NK cells in S2, inhibitory immune cells like Tregs and MDSCs might contribute to deficient cytolytic activities. Since GZMA*PRF1 could represent tumor microenvironment cytolytic activities (57), these data indicated that S1 may possess superior cytolytic activities which might eliminate tumor cells conspicuously (**Supplementary Figure 11A**). In addition, only activated CD8+ T cells were connected with survival events in two subgroups at *p* < 0.05, however, they anticipated contradicting outcomes (**Supplementary Figure 10B**, **Supplementary Table 6**). This implied that CD8+ T cells might play dual roles in neuroblastoma patients, i.e., CD8+ T cells functioned as a normal beneficial factor in malignant tumors in S2, however, impeding patients of S1 from recovering.

Then, we examined the intrinsic immune escape mechanism in neuroblastoma. Down-regulations of interferon signals and droppings of two γ-IFN receptors were observed in S2 (**Supplementary Figures 3**, **11B**), whereas IL-2 was increased in S1 which might stimulate T cell differentiation. A loss of HLA-class I/II can aid tumor cells to escape from immune monitoring. HLA-A and HLA-C were lower in S2, making the tumors prone to survive (**Supplementary Figure 11C**) (58, 59). The expression levels of *PDCD1*, *PDL1* and *CTLA4*, which are critical immune checkpoint genes, were also affected in S2 (60). Overall, these data suggest that disturbance of the immune system may be underneath the poor outcomes of the patients in S2.

## Discussion

One of the cruxes for neuroblastoma treatments is the heterogeneity. *MYCN* amplification and INSS risk classification have improved the efficacy to herald survivals, which many studies have unraveled genetic polymorphisms among. However, the current staging and grading systems are mainly based on clinical phenotypes, while it is steadily accepted that patients should be categorized by genetic associations.

Machine-learning and deep-learning methods have been used in medicine for many years. Generally, a deep-learning model receives multi-omics data and predicts outcomes by one or more neural networks. Chaudhary et al. used RNA-seq, miRNA-seq and DNA methylation data to train an autoencoder and partition patients into two prognostic groups (20). Chabon et al. sequenced SNV and CNV data of cell-free DNA in patients with lung cancers and controls. They established a ‘Lung-CLip’ machine-learning model to score each patient and determined whether a patient got lung cancer by the relative score (19). In this study, we used a DL-based classifier to significantly improve the prediction of neuroblastoma outcomes. We fed 172 genes expression data to the neural network and enrolled the attention mechanism into the survival classifier. The output probability could tell whether a patient could be dead or alive. Moreover, the 172 features selected for survival prediction could help characterize the genetic heterogeneities among the neuroblastoma patients.

A special attention mechanism was employed to combine two different parts of RNAs together (23). The attention mechanism is firstly presented by Vaswani et al. and widely used in computer vision and natural language processing (61), which is helpful to find interactions among different features, such as importance, relationship and so on. The attention mechanism can help the network learn how these two different groups of genes interact with each other. Information learned by the network can help it achieve a better performance. Indeed, our model outperformed traditional cox models, gaining a 5-year-survival AUC 0.974 and 0.896 in GSE49710 and EMTAB cohorts respectively. Besides, the PCA method failed to partition patients into appropriate prognostic groups, suggesting the superiority of our methods. Finally, we ran the gamut from all the samples in two cohorts, showing the robustness of our DL-based model.

For a long period, lncRNAs have been thought fruitless until recent advances that they might participate in chromosome stabilization, transcriptional initiation, localization, etc., thus broadening the cancer epigenetic network and making it possible for new drugs (62–64). Here, we identified four critical lncRNAs: *MYCNOS*, *TERC*, *SNHG1* and *MIR17HG*. *MYCNOS*, the antisense of *MYCN*, functions as the regulator of upstream *MYCN* promotor to enhance *MYCN* expressions. *TERC*, the telomerase RNA component, part of the telomerase, could proliferate prostate cancer cells (65, 66). *SNHG1* up-regulates in colorectal, liver, prostate and gastric cancers, which is the biomarker for decreased survivals (67). Also it contributes to the neuroinflammation in Parkinson’s disease (68). *MIR17HG* promotes colorectal as well as gastric cancer progression and up-regulates *PD-L1* expression, which could be inhibited by γ-IFN (69, 70). Investigations about those lncRNAs indicated that they could be engaged in the oncogenesis of neuroblastoma.

Our DL-based approach evinced a pathbreaking conjecture for survivals of neuroblastoma patients, still, there are some caveats should be aware of. First, neural networks are thought to be uninterpreted for now. We tried to exploit an attention mechanism to decipher underlying juxtapositions of genes involved in neuroblastoma, however, we could not declare how these neural networks work explicitly. Second, we only applied our model into two datasets that provided high-quality sequencing results as well as unequivocal labels and clinical annotations for each patient. We expected to test the reliability in more large cohorts. Last but not the least, we exerted neural networks on 172 features, which would be an obstacle for massive use in clinical examination due to its costs.

In summary, a DL-based model was constructed using 172 gene expressions to forecast survival status of neuroblastoma. Patients were split into two groups, which presented distinct microenvironments and clinical denouements. Our work paved the way for applications of artificial intelligence in medicine, not only in survival prediction, but also biological interpretations and associated accurate medicine.

## Data Availability Statement

Publicly available datasets were analyzed in this study. This data can be found here: https://www.ncbi.nlm.nih.gov/geo/query/acc.cgi?acc=GSE49710 https://wwwdev.ebi.ac.uk/arrayexpress/experiments/E-MTAB-8248/.

## Ethics Statement

This study did not need approvals by the Ethics Committee of Tongji Hospital, Wuhan, China according to the regulations. This study fully complied guidelines of GEO and ArrayExpress.

## Author Contributions

CF, TX, and ZY contributed to devising the overall pipeline. TX trained neural networks and CF conducted the following analysis. XM, XC, GH, and XZ contributed to providing biological and technical instructions. FC, BX and JF contributed to concrete guides and funding in this project. All authors contributed to the article and approved the submitted version.

## Funding

This work was supported by the National Key Research and Development Program of China [2016YFE0203900].

## Conflict of Interest

The authors declare that the research was conducted in the absence of any commercial or financial relationships that could be construed as a potential conflict of interest.

## Supplementary Material

Supplementary Figure 1 Consensus clustering results for GSE49710. **(A)** Cumulative density functions (CDF) for k=2 to 8. **(B)** Relative changes in CDF curves. **(C–F)** Consensus matrices for k=2 to 5.

Supplementary Figure 2 KM curves of the EMTAB cohort for OS **(A)** and EFS **(B)**. S1 (coralline line) and S2 (atroceruleous line) were determined by the same procedure as GSE49710 (both log-rank test *p* < 0.0001).

Supplementary Figure 3 GSEA plots showed that S1 exhibited higher biochemical activities **(A–E)** while S2 owned immune responses **(F–I)**.

Supplementary Figure 4 Explained variances of each top 10 PCA dimensions.

Supplementary Figure 5 Consensus clustering results for GSE49710 using PCA dimensions. **(A)** Cumulative density functions (CDF) for k=2 to 8. **(B)** Relative changes in CDF curves. **(C–F)** Consensus matrices for k=2 to 5.

Supplementary Figure 6 The univariable cox regression result of age, *MYCN* status, gender, stage, INSS-risk and DL-probability.

Supplementary Figure 7 Decision curve analysis for 3 models: DL-model (red), clinical covariates (palm green) and combined model (green).

Supplementary Figure 8 The ceRNA network associated with *CCNB1*, *CD9*, *MYCNOS*, *TERC*, *SNHG1* and *MIR17HG*.

Supplementary Figure 9 ESTIMATE scores for samples in GSE49710. **(A)** The total ESTIMATE scores. **(B)** Immune socres. **(C)** Stromal scores.

Supplementary Figure 10 The immune microenvironment in neuroblastoma. **(A)** Relative proportions of cell types in S1 and S2. Wilcoxon rank sum tests were used to detect differences between two subgroups (n=498). ns, not significant; *:0.05, **:0.01, ***:0.001, ****:0.0001 **(B)** Cox regressions for individual cell types in S1, S2 and the whole cohort. Dots are colored red when hazard ratios are higher than 1 and are colored blue when hazard ratios are less than 1. Also, a larger circle means a lower *p*-value.

Supplementary Figure 11 Immune microenvironment molecules. Differences between groups were examined by Wilcoxon rank sum tests. ns: not significant, *:0.05, **:0.01, ***:0.001, ****:0.0001 **(A)** S1 owned higher cytolytic activities, which were calculated by *GZMA***PRF1* (Wilcoxon rank sum tests: *p* < 0.001). **(B)** Cytokines in S1 and S2. **(C)** HLA molecules in S1 and S2. **(D)** Immune checkpoint molecules in S1 and S2.
