# Supplementary figures and images for: A Deep-Learning Model With the Attention Mechanism Could Rigorously Predict Survivals in Neuroblastoma
Source: Front Oncol. 2021 Jul 14;11:653863. doi: 10.3389/fonc.2021.653863 (PMC8317851; doi:10.3389/fonc.2021.653863)

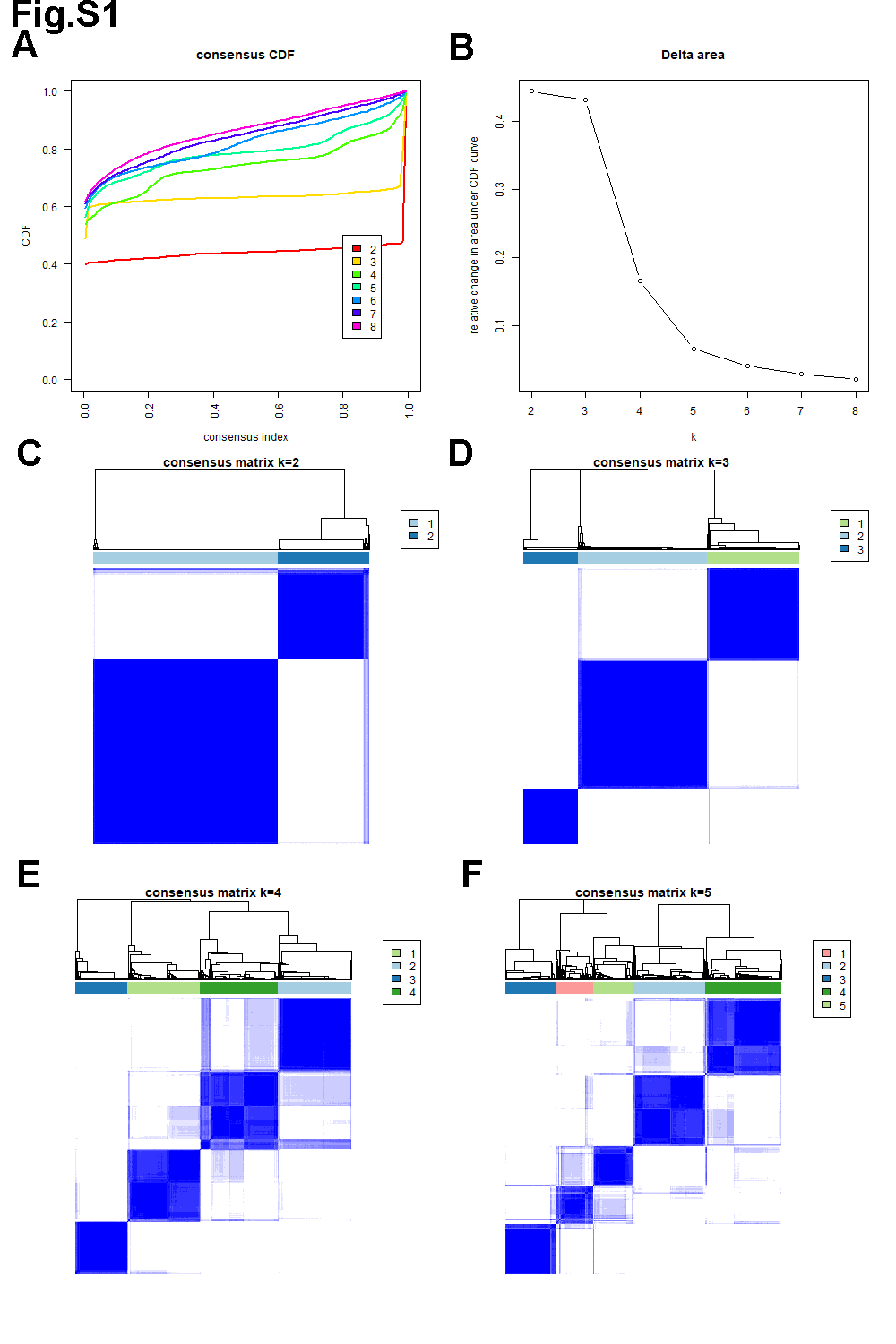

Supplement: Supplementary Figure 1 — Consensus clustering results for GSE49710. (A) Cumulative density functions (CDF) for k=2 to 8. (B) Relative changes in CDF curves. (C–F) Consensus matrices for k=2 to 5. [file Image_1.tif]

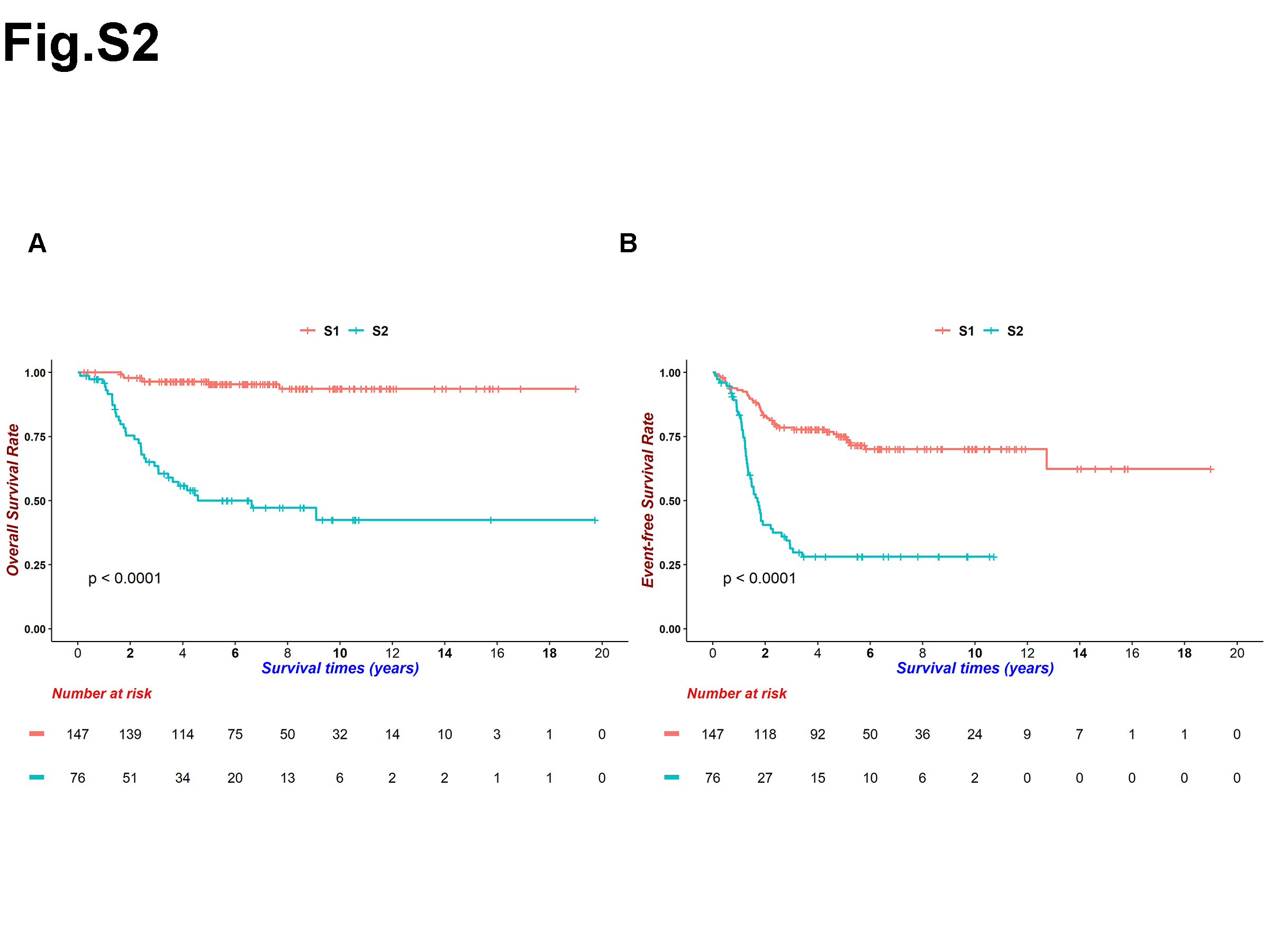

Supplement: Supplementary Figure 2 — KM curves of the EMTAB cohort for OS (A) and EFS (B). S1 (coralline line) and S2 (atroceruleous line) were determined by the same procedure as GSE49710 (both log-rank test p < 0.0001). [file Image_2.tif]

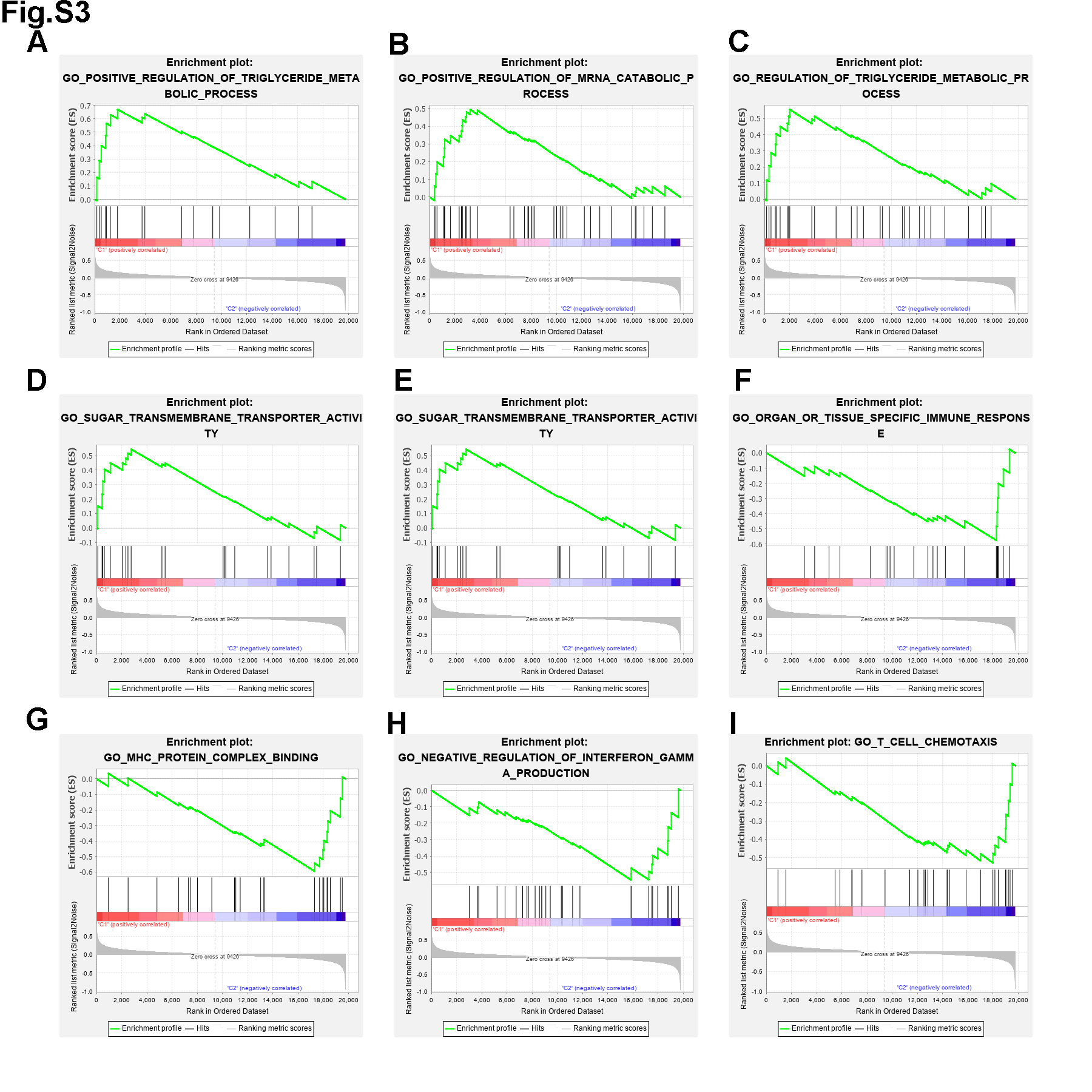

Supplement: Supplementary Figure 3 — GSEA plots showed that S1 exhibited higher biochemical activities (A–E) while S2 owned immune responses (F–I). [file Image_3.tif]

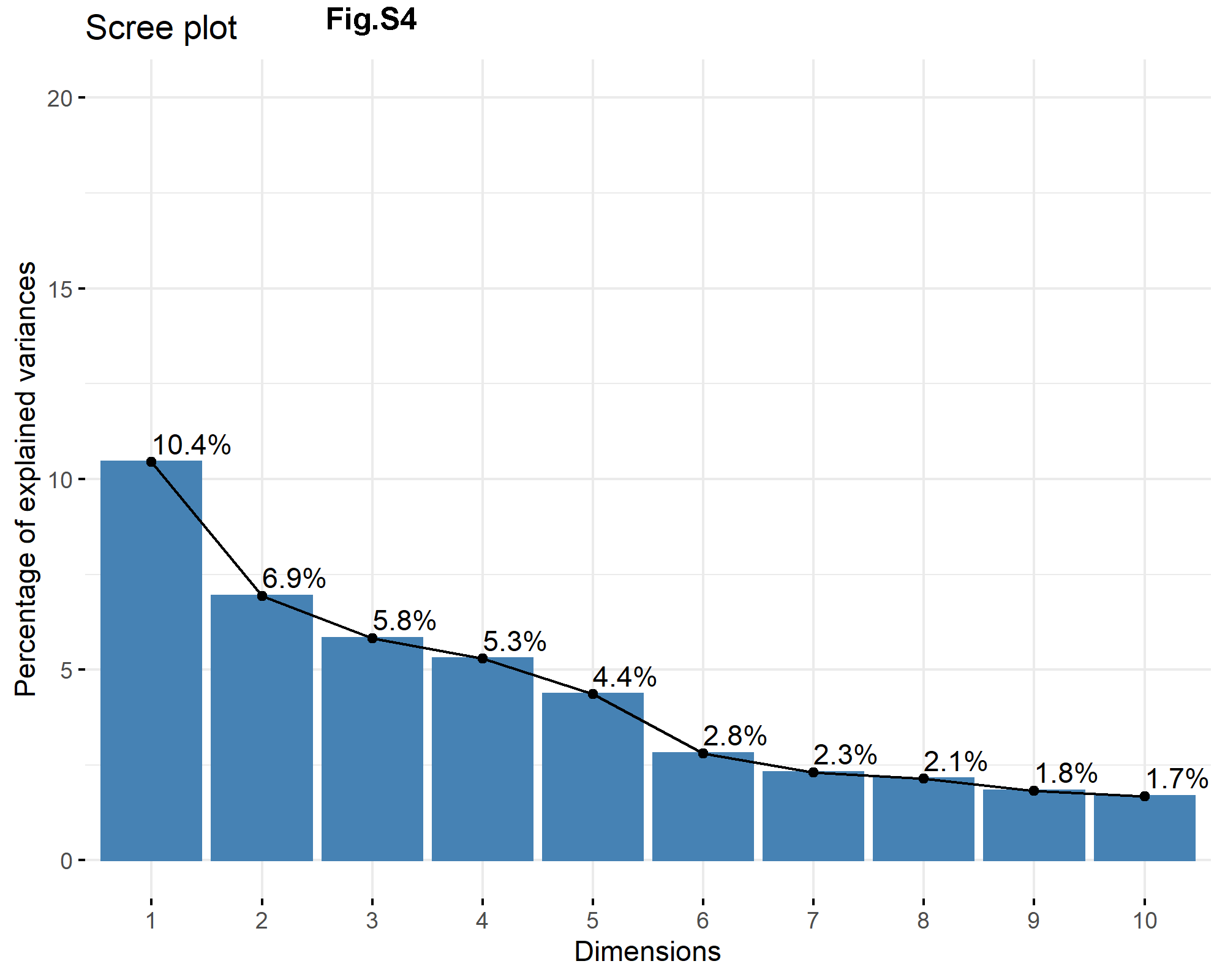

Supplement: Supplementary Figure 4 — Explained variances of each top 10 PCA dimensions. [file Image_4.tiff]

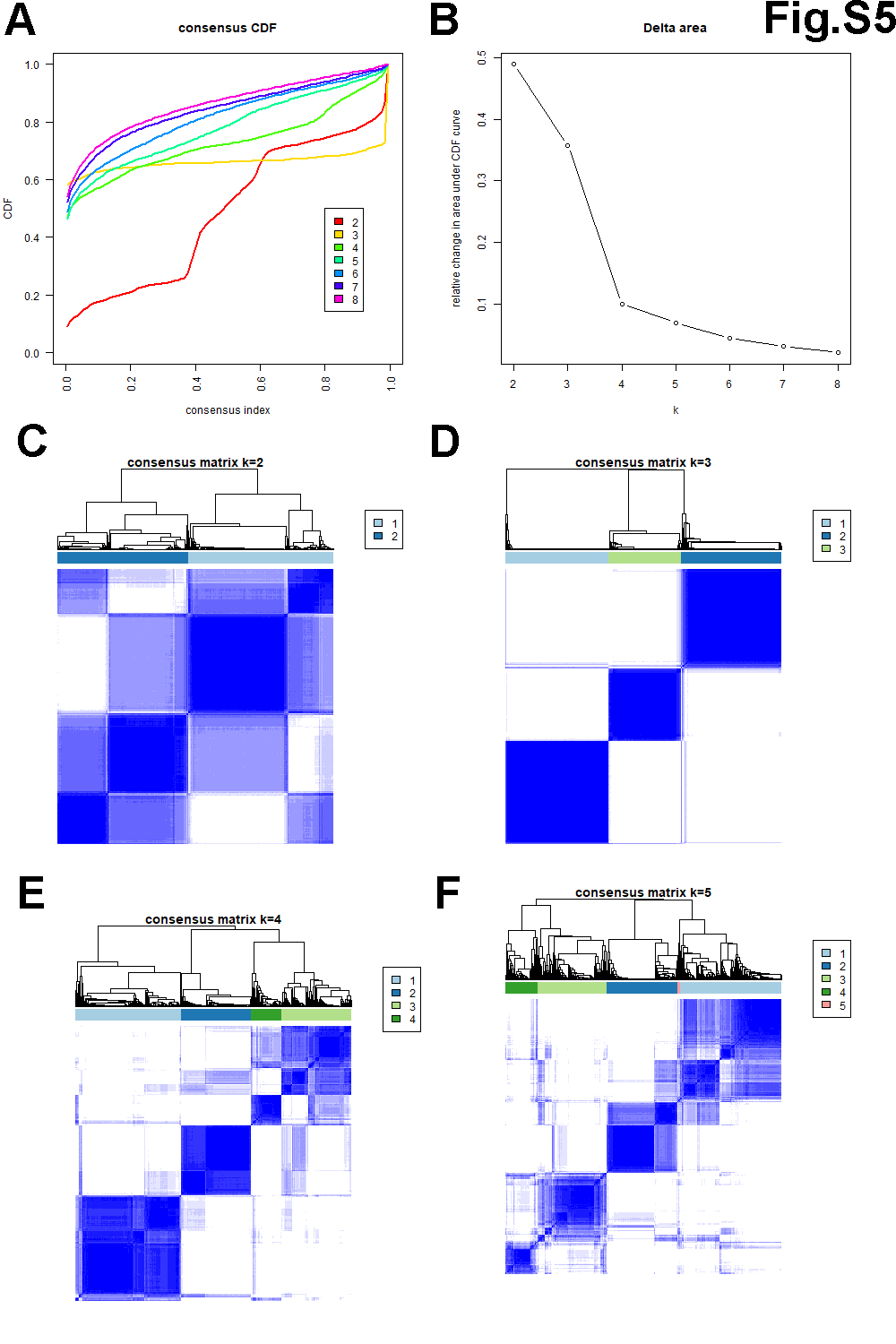

Supplement: Supplementary Figure 5 — Consensus clustering results for GSE49710 using PCA dimensions. (A) Cumulative density functions (CDF) for k=2 to 8. (B) Relative changes in CDF curves. (C–F) Consensus matrices for k=2 to 5. [file Image_5.tif]

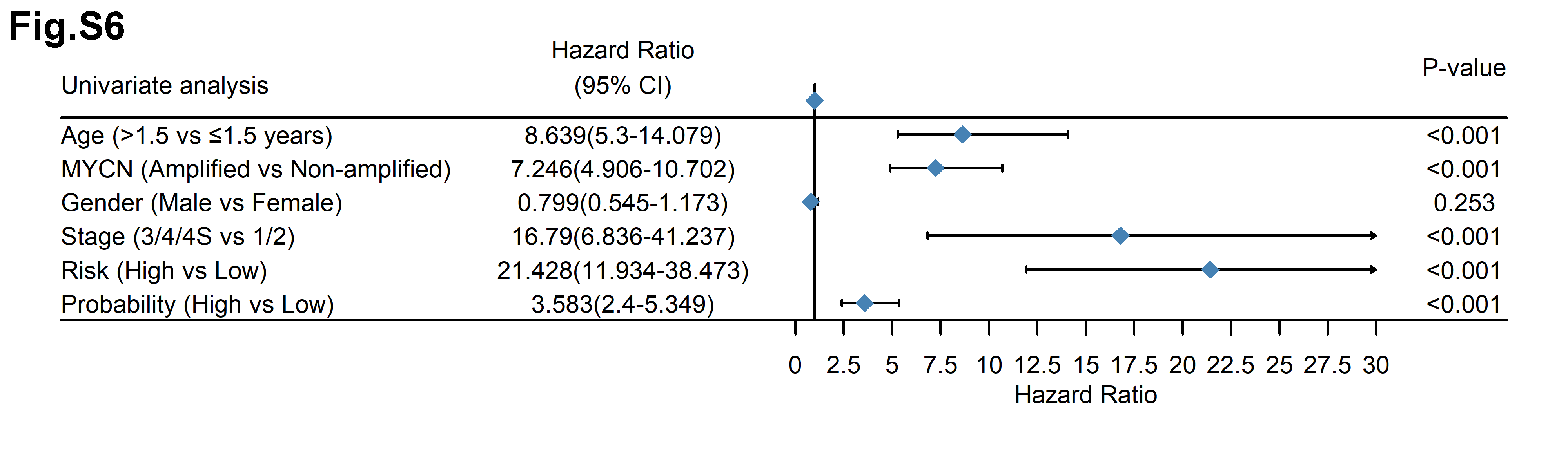

Supplement: Supplementary Figure 6 — The univariable cox regression result of age, MYCN status, gender, stage, INSS-risk and DL-probability. [file Image_6.tiff]

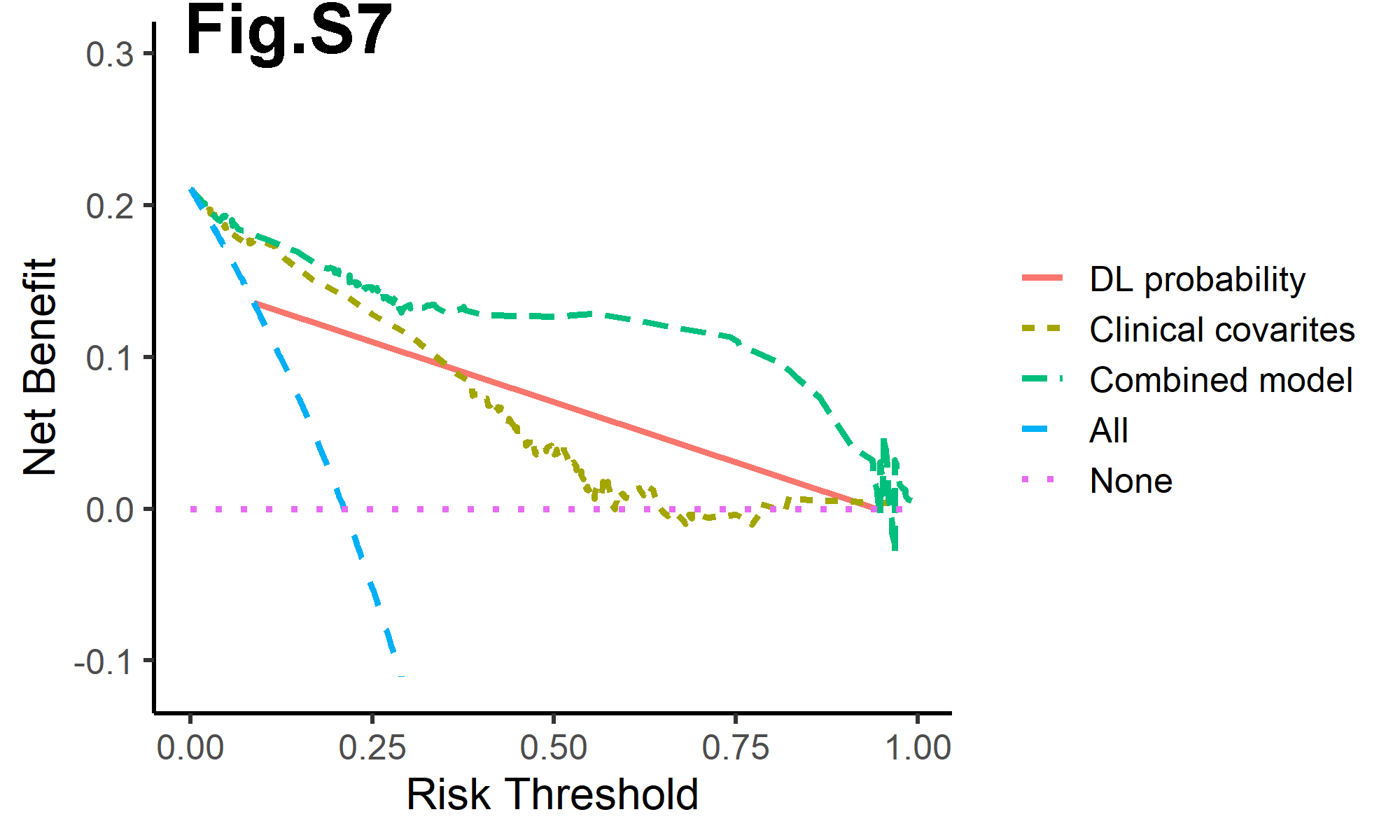

Supplement: Supplementary Figure 7 — Decision curve analysis for 3 models: DL-model (red), clinical covariates (palm green) and combined model (green). [file Image_7.tiff]

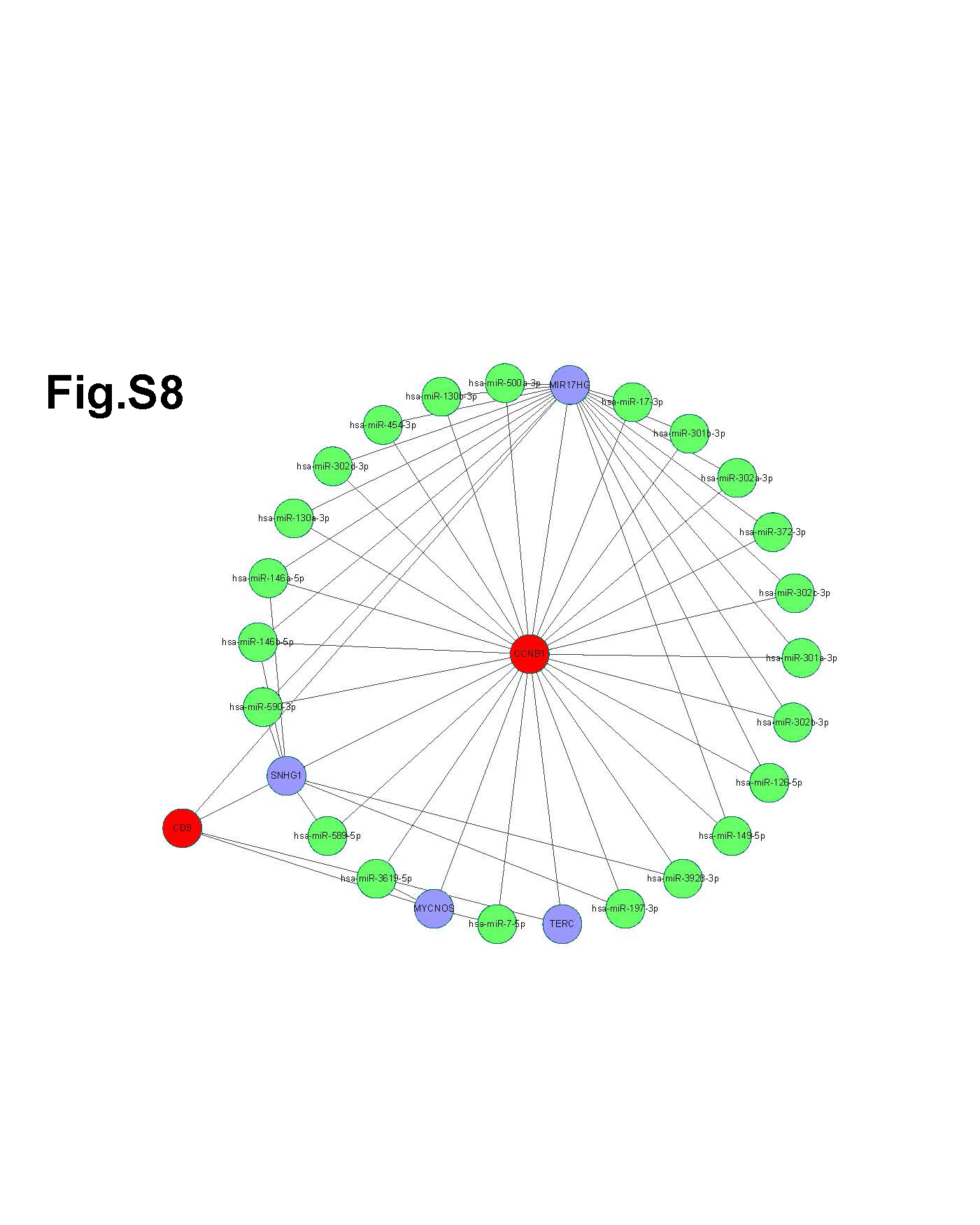

Supplement: Supplementary Figure 8 — The ceRNA network associated with CCNB1, CD9, MYCNOS, TERC, SNHG1 and MIR17HG. [file Image_8.tif]

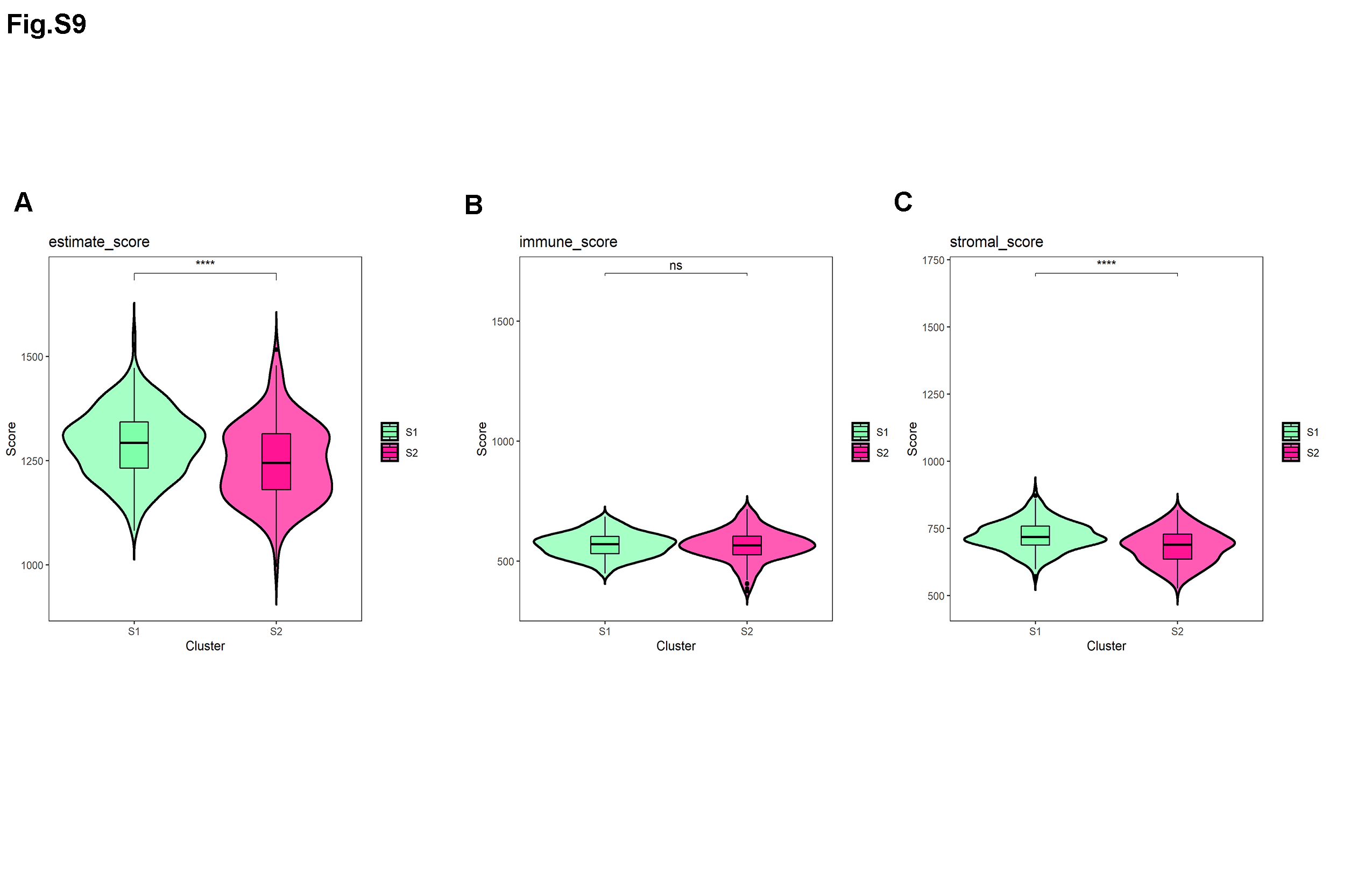

Supplement: Supplementary Figure 9 — ESTIMATE scores for samples in GSE49710. (A) The total ESTIMATE scores. (B) Immune socres. (C) Stromal scores. [file Image_9.tif]

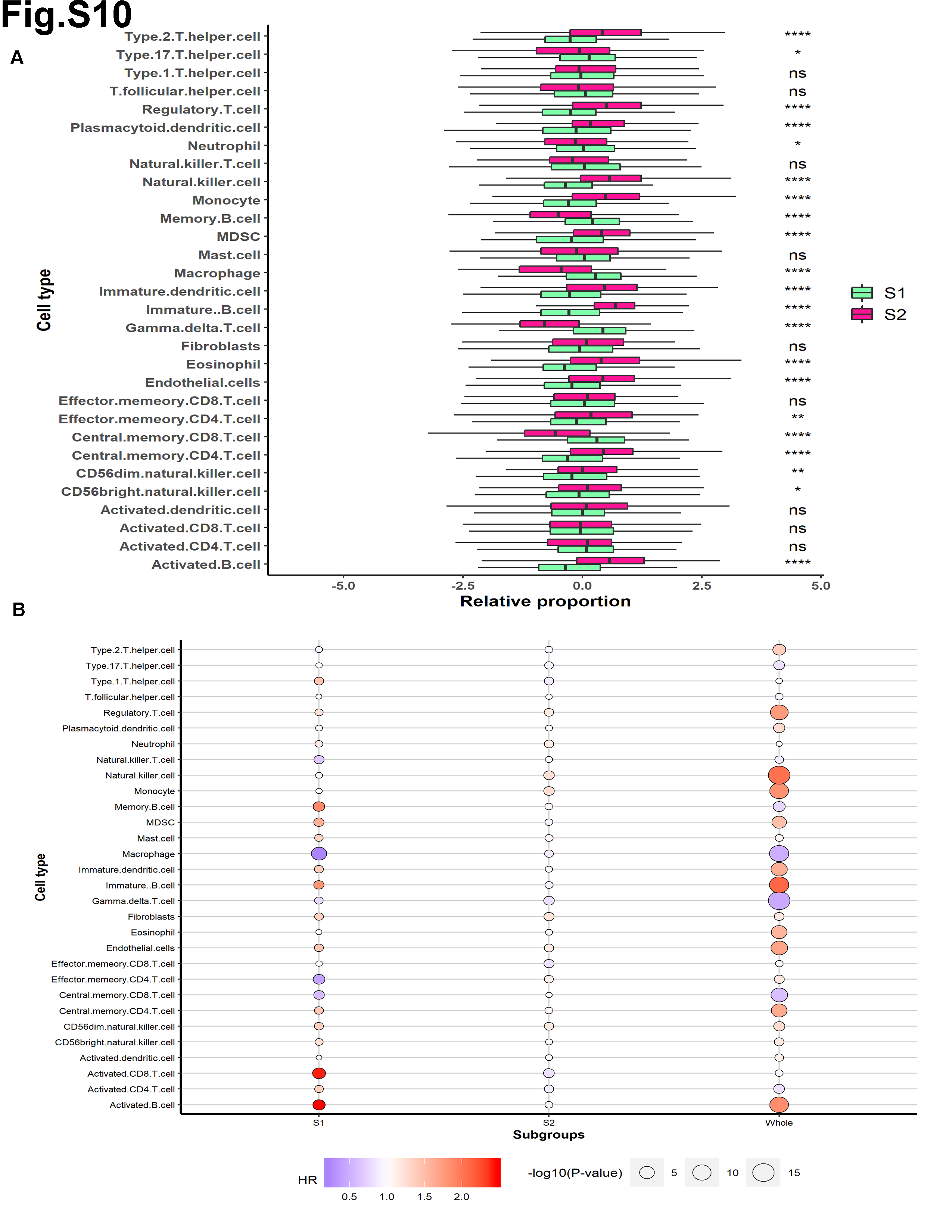

Supplement: Supplementary Figure 10 — The immune microenvironment in neuroblastoma. (A) Relative proportions of cell types in S1 and S2. Wilcoxon rank sum tests were used to detect differences between two subgroups (n=498). ns, not significant; *:0.05, **:0.01, ***:0.001, ****:0.0001 (B) Cox regressions for individual cell types in S1, S2 and the whole cohort. Dots are colored red when hazard ratios are higher than 1 and are colored blue when hazard ratios are less than 1. Also, a larger circle means a lower p-value. [file Image_10.tif]

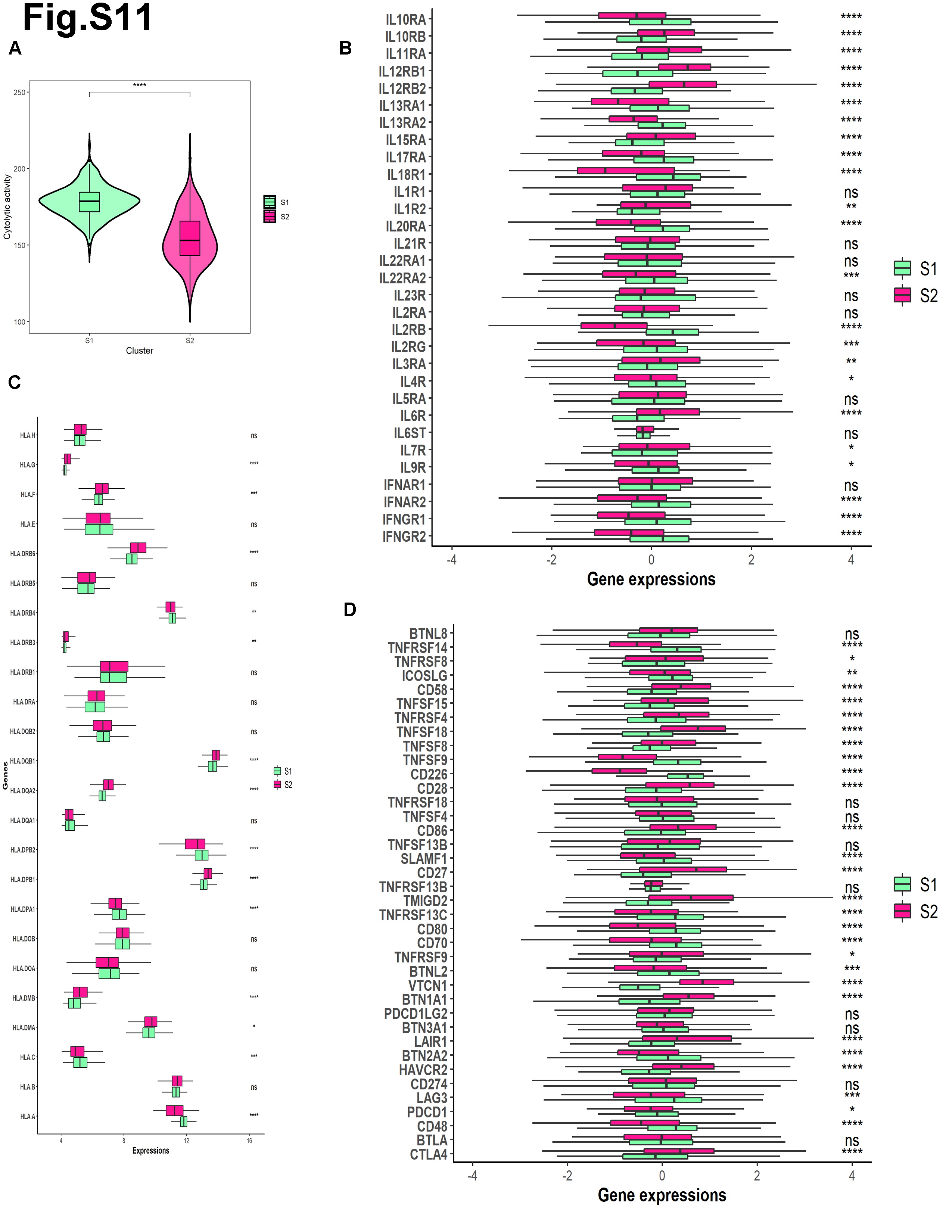

Supplement: Supplementary Figure 11 — Immune microenvironment molecules. Differences between groups were examined by Wilcoxon rank sum tests. ns: not significant, *:0.05, **:0.01, ***:0.001, ****:0.0001 (A) S1 owned higher cytolytic activities, which were calculated by GZMA*PRF1 (Wilcoxon rank sum tests: p < 0.001). (B) Cytokines in S1 and S2. (C) HLA molecules in S1 and S2. (D) Immune checkpoint molecules in S1 and S2. [file Image_11.tif]
